# Surface Modification and Characterisation of Silk Fibroin Fabric Produced by the Layer-by-Layer Self-Assembly of Multilayer Alginate/Regenerated Silk Fibroin

**DOI:** 10.1371/journal.pone.0124811

**Published:** 2015-04-28

**Authors:** Gaotian Shen, Xingyou Hu, Guoping Guan, Lu Wang

**Affiliations:** 1 Key Laboratory of Textile Science and Technology, Ministry of Education, College of Textiles, Donghua University, Songjiang District, Shanghai 201620, China; 2 Engineering Research Center of Technical Textiles, Ministry of Education, Shanghai 201620, China; Osaka University, JAPAN

## Abstract

Silk-based medical products have a long history of use as a material for surgical sutures because of their desirable mechanical properties. However, silk fibroin fabric has been reported to be haemolytic when in direct contact with blood. The layer-by-layer self-assembly technique provides a method for surface modification to improve the biocompatibility of silk fibroin fabrics. Regenerated silk fibroin and alginate, which have excellent biocompatibility and low immunogenicity, are outstanding candidates for polyelectrolyte deposition. In this study, silk fabric was degummed and positively charged to create a silk fibroin fabric that could undergo self-assembly. The multilayer self-assembly of the silk fibroin fabric was achieved by alternating the polyelectrolyte deposition of a negatively charged alginate solution (pH = 8) and a positively charged regenerated silk fibroin solution (pH = 2). Finally, the negatively charged regenerated silk fibroin solution (pH = 8) was used to assemble the outermost layer of the fabric so that the surface would be negatively charged. A stable structural transition was induced using 75% ethanol. The thickness and morphology were characterised using atomic force microscopy. The properties of the self-assembled silk fibroin fabric, such as the bursting strength, thermal stability and flushing stability, indicated that the fabric was stable. In addition, the cytocompatibility and haemocompatibility of the self-assembled silk fibroin fabrics were evaluated. The results indicated that the biocompatibility of the self-assembled multilayers was acceptable and that it improved markedly. In particular, after the self-assembly, the fabric was able to prevent platelet adhesion. Furthermore, other non-haemolytic biomaterials can be created through self-assembly of more than 1.5 bilayers, and we propose that self-assembled silk fibroin fabric may be an attractive candidate for anticoagulation applications and for promoting endothelial cell adhesion for vascular prostheses.

## Introduction

With the increase in the number of cardiovascular patients, vascular prostheses have become more desirable in recent years [1]. Synthetic vessels that are larger than 6 mm, such as poly(ethylene terephthalate) (PET) and poly(tetrafluoroethylene) (e-PTFE) grafts, are commercially available and have performed satisfactorily in clinical applications [2]. However, grafts with a diameter of less than 6 mm fail soon after introduction due to thrombus formation and intimal hyperplasia [3]. To date, there are no small-diameter vascular grafts that are approved for clinical use by the Food and Drug Administration (FDA). Hence, there is an on-going search for biomaterials with better biocompatibility and anticoagulation properties for small-diameter vascular replacements [4].

Silk fibroin (SF) fibres from *Bombyx mori* are composed of naturally produced proteins [5]. The versatility of SF fibres, along with their favourable characteristics, makes silk-based materials excellent candidates for biomedical applications [2, 6–9]. SF fibres are commonly available as sutures and have a long history of use due to their high strength and toughness [10–13]. Currently, these sutures are used in lips, eyes, and skin wounds [14]. Tristan et al. demonstrated the powerful mechanism underlying the strength of SF fibres using both computational experiments and physical experiments [15]. Additionally, regenerated silk fibroin (RSF) has been shown to be a biocompatible material [16]. In in vivo culture, RSF products induced a slight inflammatory response but did not cause fibrosis and lymphocyte invasion [17]. Asakura et al. investigated the effects of braiding, flattening, and winding the SF fibres, followed by coating them with an aqueous RSF solution. The patency (85.1%) of SF fibre grafts with an RSF coating was remarkably higher than that of e-PTFE grafts (30%) after 1 year. Endothelial cells rapidly became organised within the inner layer of the SF grafts [2, 18].

Alginate (ALG), a polysaccharide biopolymer, is a promising candidate for biotechnology applications [19–21]. ALG has been successfully used as a thickening agent, a gelling agent, and a colloidal stabiliser in cell encapsulation, drug delivery, and tissue engineering applications [20]. The high gel porosity of alginate gels allows for considerable diffusion of the mixture. Assembled multilayer ALG films can improve the stability of the modification on substrates [22]. ALG can agglomerate under normal physiological conditions [23], thereby improving the stability of polyelectrolyte layers.

For the fabrication of a uniform and stable coating on SF fabric, the layer-by-layer self-assembly technique offers an alternate strategy for surface modification [24–26]. The procedure is simple to perform and highly versatile. Alternating deposits of oppositely charged polyelectrolytes are absorbed on the matrix and form interpenetrating bilayers. The bilayers may be formed on nearly any matrix of any shape and size and generally do not require intensive chemical processing [27, 28]. The driving force for this deposition process is primarily electrostatic interactions [29, 30]. Considerable polyelectrolyte charging should be performed during the assembly. Thus, it is possible to assemble materials with desired functions to obtain silk-based materials with the appropriate properties by controlling the charging solution [31].

In this study, an RSF solution (pH<7) and an ALG solution (pH>7) were used as oppositely charged polyelectrolytes for layer-by-layer self-assembly. The pH of the polyelectrolytes must be adjusted to the appropriate value to obtain cationic and anionic polyelectrolyte solutions for the self-assembly based on the zeta potential. The objective of this study was to prepare an ALG/RSF multilayer self-assembled SF fabric with excellent cell attachment and anticoagulation properties for use as a vascular graft.

This study is the first to demonstrate the assembly of ALG and RSF on SF fabric using the layer-by-layer self-assembly technique to achieve cell adhesion and anticoagulation for vascular grafts. To achieve this goal, we ensured that the outermost layer was composed of negatively charged RSF because negatively charged platelets would not adsorb onto a surface that has the same charge [32] and because RSF is conducive to endothelial cell adhesion [33].

## Materials and Methods

### 2.1 Materials

**Fig 1:**
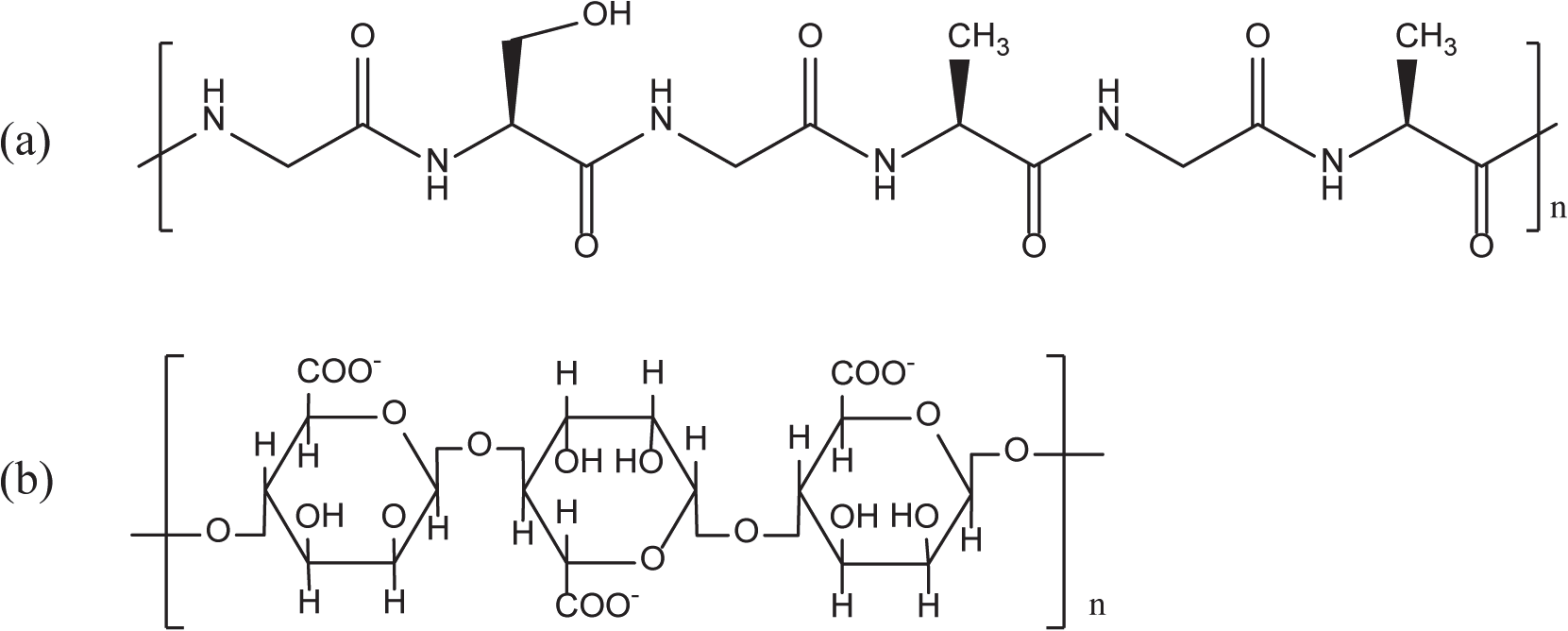
Chemical structure of the polyelectrolytes used. (a) Silk Fibroin, (b) Alginate.

Natural silk filaments were provided by Hangzhou Qiantang Silk Company Ltd, Zhejiang, China. The filaments were woven to obtain a 1/1 natural silk fabric. Sodium carbonate (Na_2_CO_3_) was purchased as a powder from Sinopharm Chemical Reagent Company Ltd, Shanghai, China. The natural silk fabric was degummed 3 times using a 0.05 wt.% Na_2_CO_3_ solution at 98°C to obtain the SF fabric. Phosphate-buffered saline (PBS) was purchased from the Solarbio Technology Company Ltd, Beijing, China. Alginate (ALG) was purchased as a powder from Sinopharm Chemical Reagent Company Ltd, Shanghai, China. The RSF solution was derived from *Bombyx mori* cocoons that were dissolved and dialysed (molecular weight cut off 2000) in distilled water for 48 h. Then, the RSF solution was dialysed against a 20 wt.% poly(ethylene glycol) solution (20000 g/mol) for 30 min. The polyelectrolytes were used as received without further purification and were prepared as 1 g/L solutions. Silicon wafers were purchased from Ruicai Technology Company Ltd, Suzhou, China. (3-Aminopropyl) triethoxysilane (APTES) was used to form an amination surface on a silicon wafer purchased from Sigma-Aldrich. The chemical structures of the basic materials used to perform the experiments are shown in Fig 1.

### 2.2 Zeta potential measurements

The zeta potential was measured to determine the charge of the RSF solution and the ALG solution over the pH range 2–11 [34]. The concentration of the RSF and ALG solutions was 1 g/L, and the pH of the solutions was adjusted using hydrochloric acid and sodium hydroxide. Measurements were performed using Malvern nano-ZS equipment (Malvern Instruments, England). Each value of the zeta potential was obtained at ambient conditions as the average of three independent measurements of 10 sub-runs each. The RSF solution tended to gel rapidly under acidic conditions; therefore, it was first prepared in a 1 g/L neutral solution, and the pH was decreased before each measurement.

### 2.3 Fabrication of ALG/RSF multilayers

**Fig 2:**
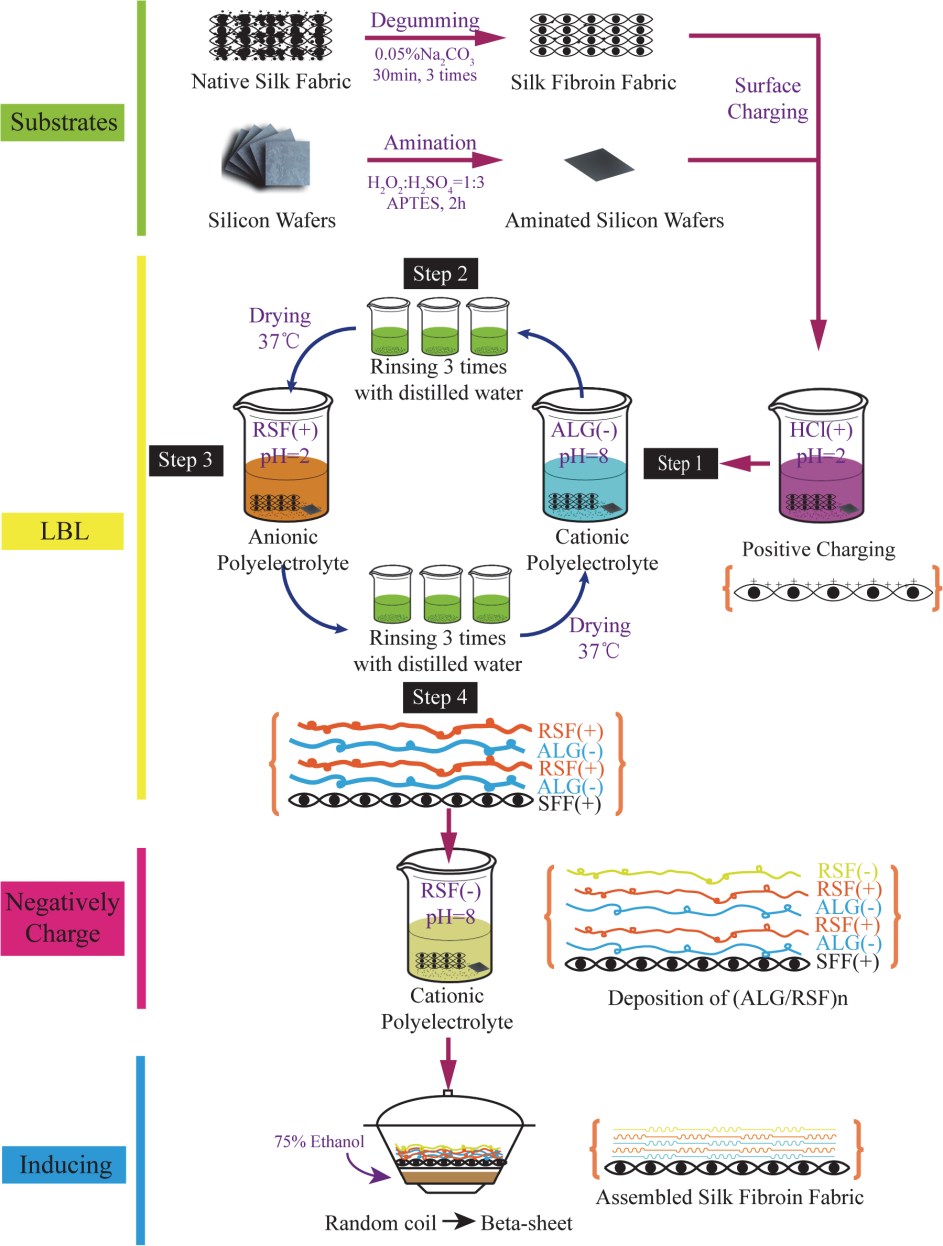
Diagram showing the deposition of ALG and RSF to create 1.5 to 9.5 polyelectrolyte bilayers on SF fabric followed by 75% ethanol treatment.

A positively charged SF fabric was prepared by immersing the SF fabric into HCl (pH = 2) for 20 min. The layer-by-layer composite SF fabric was generated by alternating the adsorption of positively charged RSF and negatively charged ALG solutions on the positively charged SF fabric. The negatively charged RSF formed the outermost layer of the assembly. To characterise the assembly of the ALG/RSF multilayers, silicon wafers were utilised for deposition simultaneously with the layer-by-layer self-assembly process. Before the assembly, the silicon wafers were treated using APTES to form an amination surface that simulated the SF fabric. The fabrication process is shown in Fig 2. The 1.5 to 9.5 polyelectrolyte bilayers on the surface of the SF fabrics were assembled by alternately dipping the substrate into solutions of ALG and RSF [35]. First, the SF fabrics were immersed in HCl (pH = 2) for 20 min. Then, the positively charged SF fabrics were immersed in the ALG solution for 20 min and rinsed in a distilled water bath for 2 min. Second, the fabrics were dried at 37°C for 30 min. Then, the fabrics were immersed in the RSF solution for 20 min, followed by the same rinsing and drying procedures. The adsorption, rinsing and drying steps were repeated until the desired number of deposition bilayers was attained. Finally, the fabrics were immersed in an alkaline RSF solution (pH>7) for 20 min for the assembly of the outermost layer. Here, (ALG/RSF)_n_ was used as the formula to describe the layer-by-layer structured SF fabrics. The values of n were 1.5, 3.5, 5.5, 7.5, and 9.5, and the outermost layer was negatively charged RSF. The assembled SF fabrics could be stabilised by a 24-h treatment with ethanol, which induces a transition from random coils to a beta-sheet structure [36, 37].

### 2.4 Characterisation techniques

Fourier transformed infrared spectroscopy of the SF fabric and the (ALG/RSF)_n_ self-assembled SF fabric was conducted on a Nicolet 6700 (Thermofisher) FTIR spectrophotometer fitted with an attenuated total reflectance (ATR) attachment for scanning in the range of 750–4000 cm^-1^. The surface morphology and topography of the silicon wafers and self-assembled SF fabrics were observed by atomic force microscopy (AFM) using an SPM NanoScope IV instrument (Veeco Instruments). The thickness of the self-assembled multilayers on silicon wafers was measured for the removal of each layer from the surface of the silicon wafers. The distance from the surface to the silicon wafer was measured and calculated. The curve length changed as the surface roughness changed. The roughness index (RI) of the multilayers was calculated using Eq (1) as an indirect measure of the evenness. The self-assembled multilayers on SF fabrics were characterised by thermogravimetric analysis (TGA) using a TG209F1 (Germany) in nitrogen atmosphere with a heating rate of 20.0°C/min. Then, 5 mg of fabric was heated from 0 to 900°C, and the mass loss was recorded. Five repeated measurements were performed at different sites on each silicon wafer and self-assembled fabric. All measurements were performed under ambient conditions.

$$Roughness\;index(RI)=\frac{Curve\;length\;of\;bilayers'\;surface}{Straight\;stretch\;of\;the\;curve}$$ (1)

### 2.5 Flushing stability

**Fig 3:**
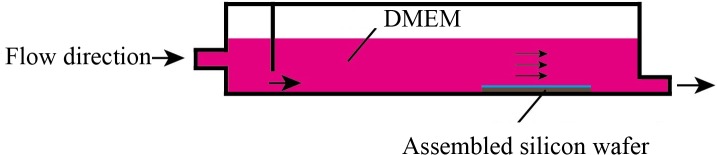
Laminar flow system designed to simulate blood flow.

Because the (ALG/RSF)_n_ self-assembled SF fabric may be used as vascular graft, the multilayers would be subjected to blood flow in vivo. Therefore, we evaluated their stability using a laminar flow system designed to simulate the blood flow in the arteries where the self-assembled material would be used (Fig 3). The system of silicon wafers that were self-assembled with (ALG/RSF)_n_ multilayers after 75% ethonal treatment was flushed at 37°C for 24 h. The shear stress was controlled to 15 dyn/cm^2^, which is equal to the stress in an artery in vivo [38, 39].

### 2.6 Bursting strength

The bursting strength test was performed in a universal testing system (YG-B 026G-500, Darong, China). Five round specimens that were 4 cm in diameter were used for the test and were mounted between two circular clamps. A probe with a 1.5-mm diameter passed through the centre of the clamps and specimens. A moving rate of 50 mm/min was applied during the testing process. The ultimate tensile strength (UTS) was defined as the highest recorded stress that the specimens withstood prior to failure. The estimated bursting strength was calculated from the UTS using the following Eq (2).

$$Bursting\;strength=\frac{UTS}{\pi r^{2}}$$ (2)

where *UTS* is the maximum breaking force, and *r* is the radius of the probe (0.75 mm in this test).

### 2.7 Cell viability and proliferation assays

Porcine iliac artery endothelial cells (PIECs) were supplied by Keygen Biotechnology Company and cultured in Dulbecco’s modified Eagle’s medium (DMEM) (Gibco) supplemented with 10% foetal calf serum (Gibco) and 1% penicillin-streptomycin (Gibco). The cells were cultured at 37°C in a 5% CO_2_ incubator. The medium was changed every other day. The samples were cut into discs that were 1.5 cm in diameter to cover the bottom of 24-well plates. The PIECs were seeded into the test wells at a density of approximately 5×10^3^ cells/well.

Cell proliferation was assessed using cell counting kit-8 (CCK-8). The cells were cultured for 1, 3, 5, and 7 days, after which the samples were mixed with 360 μL of medium and 40 μL of CCK-8 solution and incubated at 37°C to form formazan. After 4 h, the optical density (OD) at 450 nm was measured using a microplate reader (Multiskan FC, Thermo). Three parallel replicates were evaluated for each sample.

After 1 or 5 days, the PIECs were seeded onto the SF fabric, (ALG/RSF)_1.5_ or (ALG/RSF)_9.5_ and were stained using phalloidin-TRITC and 4',6-diamidino-2-phenylindole (DAPI). First, the cells were fixed using a 2.5% glutaraldehyde solution at 4°C for 1 h and then rinsed 3 times with PBS. Then, the PIECs and fabrics were stained with phalloidin-TRITC solution for 30 min at room temperature. Afterward, the samples were rinsed 3 times with PBS and followed staining with DAPI solution for 10 min at room temperature. The stained samples were stored in glycerol until use. Confocal images were obtained using a confocal laser scanning microscope (Lavision, Germany).

### 2.8 Platelet adhesion test

In our previous studies [40], a haemocompatibility assay was conducted. Human whole blood with a 3.8% sodium citrate solution was provided by Shanghai General Hospital (Shanghai, China). The whole blood was centrifuged at 1500 rpm for 15 min to obtain platelet-rich plasma (PRP) and was centrifuged at 3000 rpm for 10 min to obtain platelet-poor plasma (PPP) using a Thermo Scientific Biofuge Primo R (Thermo Fisher Scientific, China). The density of the platelets in the PRP was adjusted to 1×10^5^ cells/μL by mixing the PRP and the PPP. The platelet concentration was determined using a haematology analyser (Qiujing Biochemistry Ltd, China). Samples were placed in 24-well plates with 200 μL of the platelet suspension and then incubated for 3 hours at 37°C. After 3 washes with PBS, the adhered platelets were fixed using 2.5% glutaraldehyde in PBS for 60 min at 4°C and then washed with PBS and dehydrated with 30, 50, 60, 70, 80, 90 and 100% ethanol solutions in sequence. The samples were dried and then sputter-coated for SEM observation.

### 2.9 Haemolytic assay

Human whole blood was centrifuged and washed five times with PBS to remove the serum. The separated human red blood cells were diluted 35 times in PBS solution to obtain human red blood cells (HRBCs). Then, 1 mL of the HRBC suspension was transferred to a 10-mL Eppendorf tube, which was filled with either 4 mL of distilled water as the positive control or PBS buffer as the negative control. Both the SF fabric and the (ALG/RSF)_n_ self-assembled SF fabric (2 cm × 2 cm) were incubated in a suspension containing 1 mL of HRBCs and 4 mL of PBS buffer at 37°C for 2 h and were then centrifuged (5000 rpm, 3 min). Then, the optical density of the supernatant was determined using a Perkin Elmer Lambda 25 UV-visible spectrophotometer operating at 540 nm. The haemolysis percentage (HP) was calculated using Eq (3).

$$HP(\%)=\frac{\left(D_{t}-D_{nc}\right)}{\left(D_{pc}-D_{nc}\right)}\times 100\%$$ (3)

where *D*_*t*_ is the absorbance of the test sample, and *D*_*pc*_ and *D*_*nc*_ are the absorbance of the positive and negative controls, respectively.

### 2.10 Ethics statement

Cells were obtained from the Keygen Biotechnology Company. The human whole blood collection for this study was approved by the Shanghai First People’s Hospital (Shanghai, China) ethics committee, and written informed consent was obtained from all donors prior to phlebotomy.

### 2.11 Statistical analysis

The data are reported as the means and standard deviations, and the error bars in the figures correspond to one standard deviation. All the statistical analyses were performed using the one-way analysis of variance (ANOVA). A *p* value < 0.05 was selected as the confidence interval when differences were first found to be significant. The data in the tables are marked by (*) for *p* < 0.05 and (**) for *p* < 0.01.

## Results and Discussion

### 3.1 Zeta potential of polyelectrolytes

The stability of polyelectrolyte solutions in part depends on the charge of the dispersed phase. Particles with zeta potentials less than ±5 mV are considered highly unstable, and rapid aggregation will occur under this condition [41]. Conversely, the stability of polyelectrolyte systems increases at higher zeta potentials.

**Fig 4:**
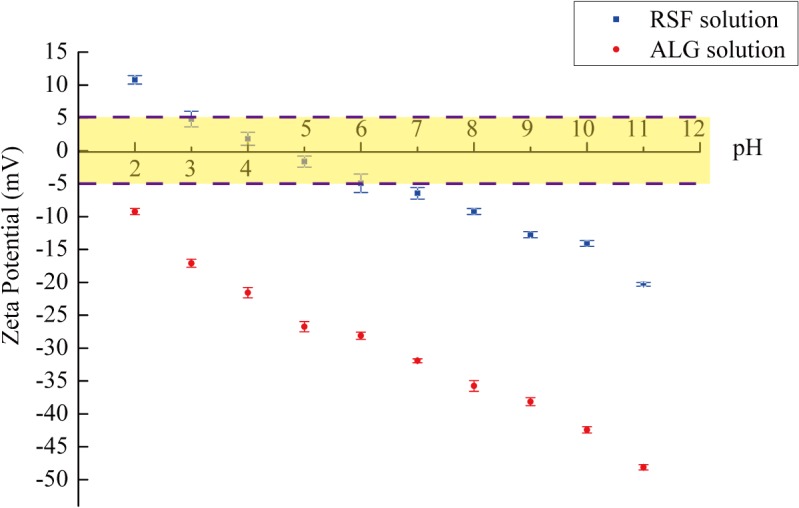
Relationship between the charges of the polyelectrolyte solutions and the pH values. Zeta potential measurements for blue label RSF solution, red label ALG solution.

Fig 4 shows the relationship between the zeta potential and pH values of the RSF and ALG solutions. The zeta potential values of the polyelectrolytes changed with the pH of the solutions. Silk fibroin proteins mainly consist of the neutral amino acids glycine, alanine, and serine with a few acidic residues (Fig 1(A)). The zeta potential measurements show that silk fibroin has an isoelectric point just between pH 4–5 and that silk fibroin is highly unstable at pH values from 3–7. The zeta potential is 10.8±0.64 mV when pH 2, which is suitable for cationic polyelectrolytes. The zeta potential is -9.23±0.46 mV when pH 8, which is suitable for anionic polyelectrolytes. The zeta potential of the alginate solution became negative even at pH 2 and became more negative with increasing pH. The high fraction of dissociated carboxyl groups in the alginate chain contributed to the high zeta potential and high electrostatic repulsion (Fig 1(B)) [42]. The ALG solution should be selected as an anionic polyelectrolyte for pH values >7. At the same time, the high stability of the environment did not change as the pH increased. Thus, the ALG solution with pH 8 was selected as the anionic polyelectrolyte in this study.

### 3.2 ATR-FTIR

**Fig 5:**
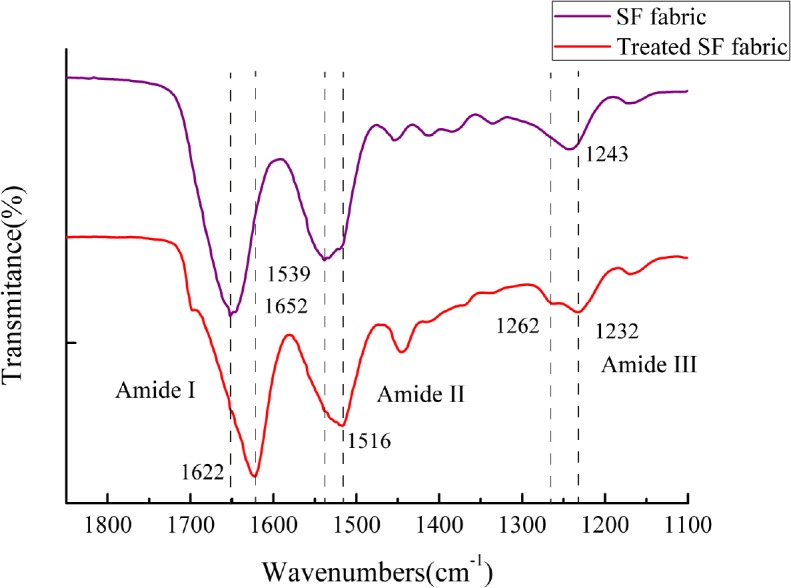
ATR-FTIR spectra of SF fabric and self-assembled (ALG/RSF)_9.5_ SF fabric treated using 75% ethanol.

ATR-FTIR is commonly used to investigate the conformation of SF fabric because the IR spectrum includes the common absorption bands that are sensitive to the molecular conformation of RSF. The results obtained for the SF fabric and the (ALG/RSF)_9.5_ self-assembled SF fabric with 75% ethanol are shown in Fig 5. The RSF in the SF fabric presented typical random coil conformations and α-chains with peaks at 1652, 1539 and 1243 cm^-1^ corresponding to amides I, II and III, respectively. The treatment with 75% ethanol for 24 h induced β-sheet transitions in the silk fibroin. This process was found to be effective at inducing conformational transitions with sharp signature peaks at 1622, 1516 and 1262 cm^-1^ for amides I, II and III, respectively, which are all related to the β-sheet conformation of the silk protein [13, 43–45].

### 3.3 Surface morphology and thickness of multilayers

**Fig 6:**
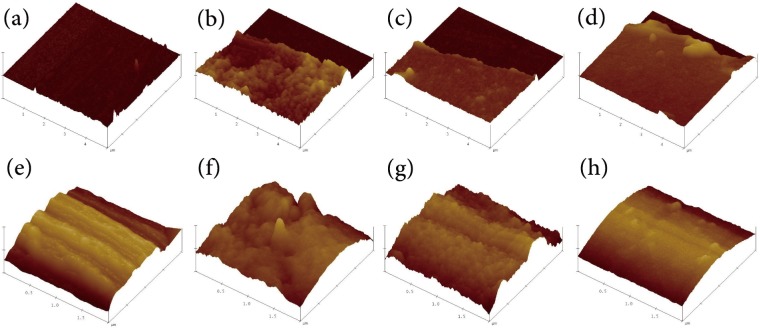
AFM images of silicon wafers and fabrics. (a) Untreated silicon wafer, (b) (ALG/RSF)_**1.5**_ silicon wafer, (c) (ALG/RSF)_**5.5**_ silicon wafer, (d) (ALG/RSF)_**9.5**_ silicon wafer, (e) SF fabric, (f) (ALG/RSF)_**1.5**_ SF fabric, (g) (ALG/RSF)_**5.5**_ SF fabric, and (h) (ALG/RSF)_**9.5**_ SF fabric.

The morphology and topography of the silicon wafers and fabrics were observed using AFM. As shown in Fig 6, the average sizes were 1 μm (silicon wafers) and 0.5 μm (fabrics). All of the silicon wafers were slashed using pins, and AFM measurements were performed for both of the multilayers and the silicon matrix. At the edge, the assembled material was squeezed and arched slightly. The yellow zone on the left corresponds to the assembled multilayers, and the dark zone on the right corresponds to the silicon matrix.

The surface topography of the silicon wafers and fabrics appeared to change qualitatively as a result of the layer-by-layer self-assembly. Before the assembly, the top view of the silicon wafer and SF fabric surface revealed a relatively smooth appearance (Fig 6(A) and 6(E)). The impurities were removed by rinsing and degumming. The grooves of the SF fabric were exposed by the degumming. After the assembly of 1.5 bilayers, the RSF and ALG were deposited on the surface of the silicon wafer and SF fabric. The morphology of the silicon wafers and the SF fabric became more uneven with many convex protrusions (Fig 6(B) and 6(F)). Furthermore, the ALG and RSF were distributed unevenly. As the number of assembled bilayers (n = 5.5) increased, the surface became smooth again compared with that of the assembled (ALG/RSF)_1.5_ SF fabric (Fig 6(C) and 6(G)). After the assembly of 9.5 bilayers, the depositions were distributed uniformly.

**Fig 7:**
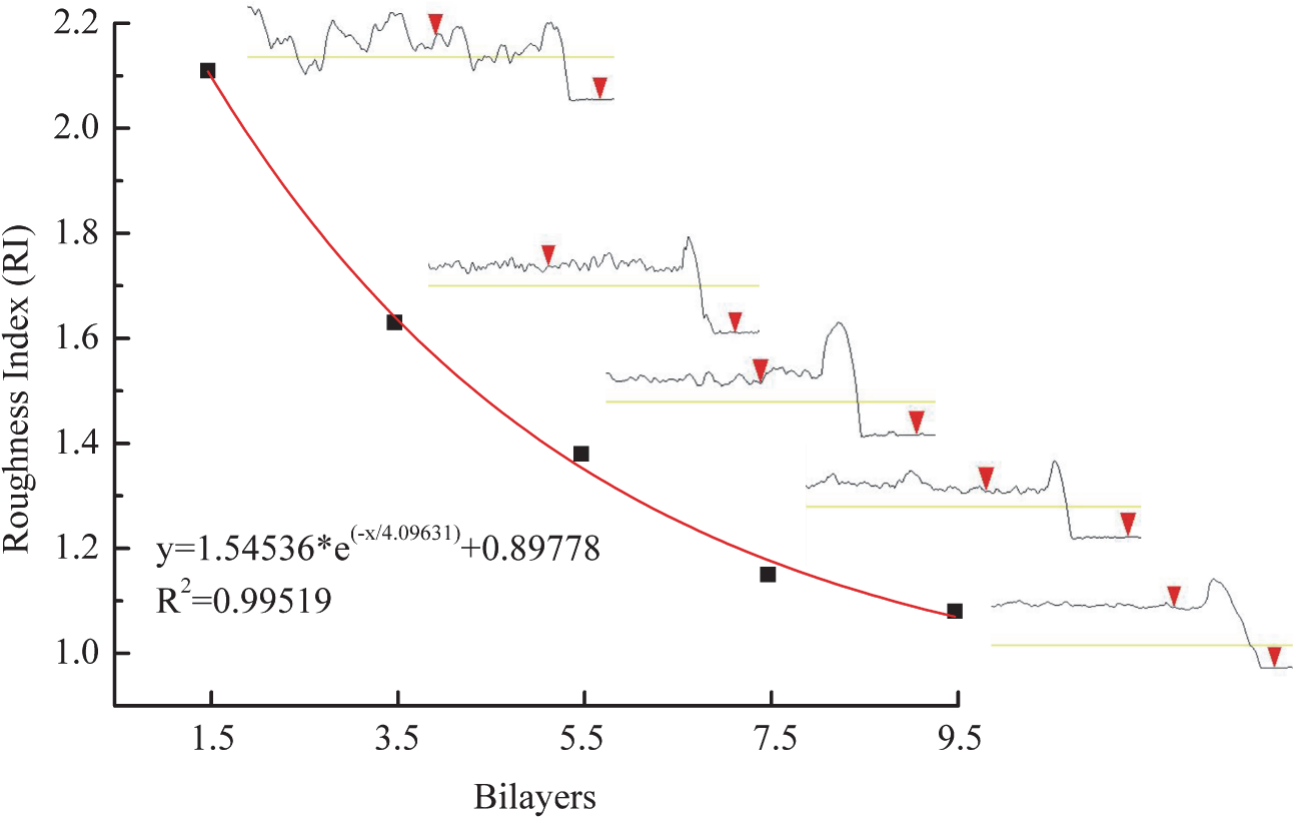
The RI and surface curve of the (ALG/RSF)_n_ fabrics self-assembled layer-by-layer on silicon wafers.

Fig 7 shows the RI and cross section of the self-assembled bilayers on silicon wafers. The results indicated that the morphology of the assembled material became smooth as the number of bilayers increased. For the first 1.5 bilayers, the altitude difference between the peaks and valleys was large and led to an RI of approximately 2.11. There was a marked drop in the RI to 1.64 after the assembly of 3.5 bilayers. The roughness clearly became smooth after the deposition of 5.5 and 7.5 bilayers; the RI declined slightly to 1.38 and 1.15, respectively. With continuing layer-by-layer self-assembly, the RI closely approached the theoretical minimum value. Moreover, the roughness closely followed an exponential trend. The difference of peak/valley (2×2 μm) is listed in Table 1. The results change from 52.52 nm for (ALG/RSF)_1.5_ to 6.25 nm for (ALG/RSF)_9.5_, indicating a smoother surface with the increasing of assembled layers.

**Table 1:** The difference of peak/valley on (ALG/RSF)_n_ silicon wafers.

Sample	(ALG/RSF)_1.5_	(ALG/RSF)_3.5_	(ALG/RSF)_5.5_	(ALG/RSF)_7.5_	(ALG/RSF)_9.5_
Difference (nm)	52.52	33.29	26.36	16.12	6.25

**Fig 8:**
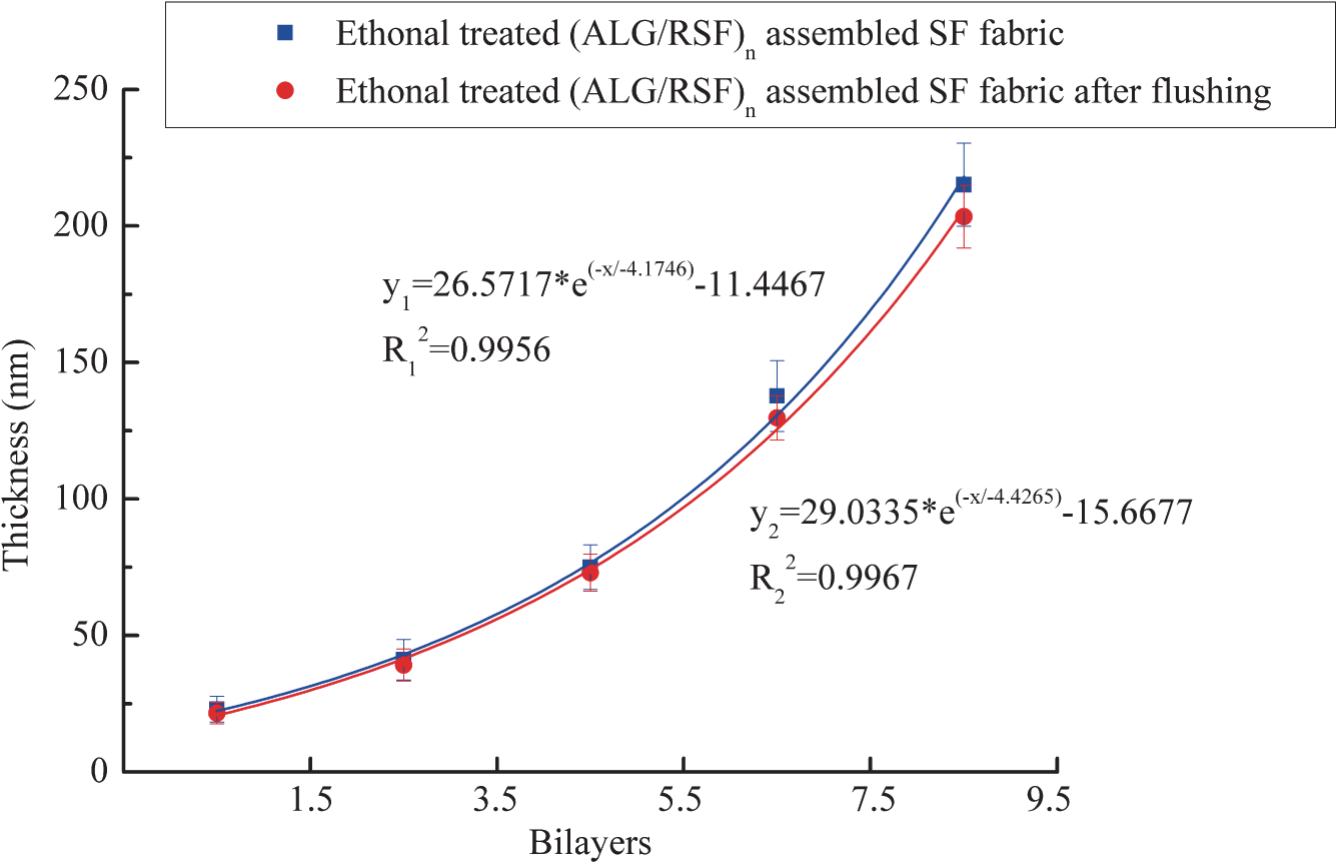
The thickness of the self-assembled (ALG/RSF)_n_ layers on silicon wafers. Blue label represents the unflushed thickness, and red label represents the flushed thickness.

The thickness of the multilayers was calculated using AFM. The results are presented in Fig 8. The (ALG/RSF)_n_ deposition was nano-sized, and the thickness of (ALG/RSF)_1.5_ was only 23 nm. As the number of self-assembled bilayers increased, the thickness of the deposition increased exponentially.

### 3.4 Thermal analysis of SF fabric and self-assembled SF fabric

**Fig 9:**
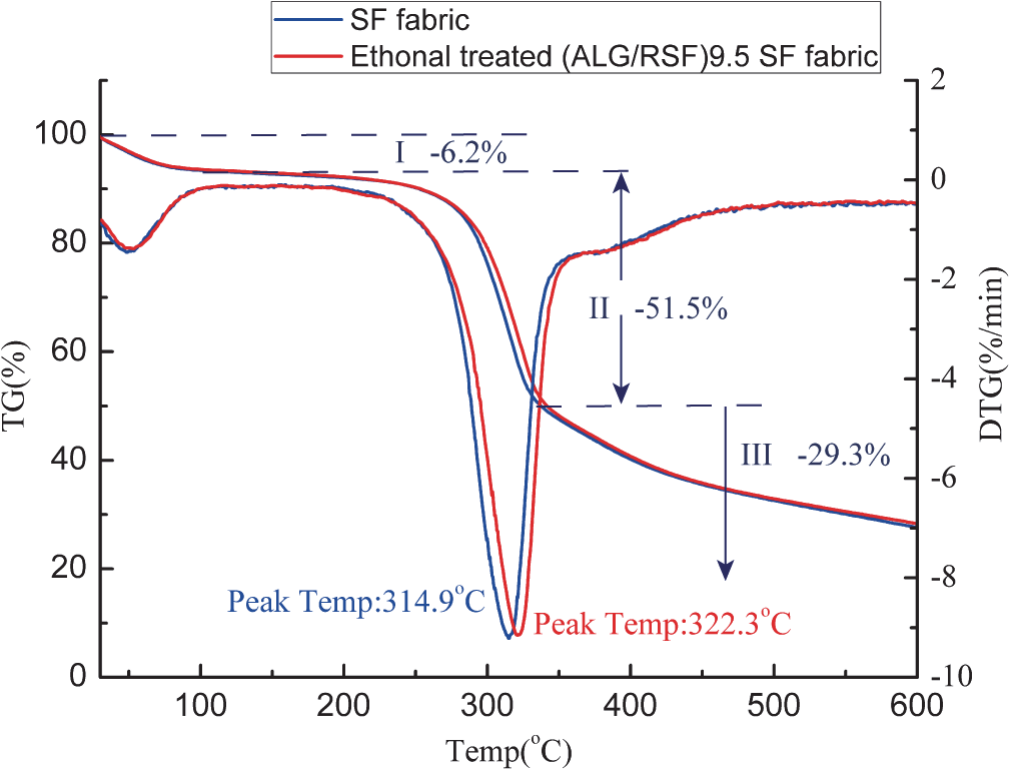
TG and DTG of SF fabric and self-assembled (ALG/RSF)_9.5_ silk fibroin fabric.

Fig 9 shows the TG and DTG of the SF fabric and the self-assembled (ALG/RSF)_9.5_ SF fabric. The transition temperature of pure alginate is 210–270°C [46]. The deposition of a small amount of ALG did not produce any evident change in the self-assembled (ALG/RSF)_9.5_ SF fabric. At area I, the mass loss percentage was 6.2% and was caused by the evaporation of water. This area did not differ between the SF fabric and the self-assembled (ALG/RSF)_9.5_ SF fabric. At area II, the mass loss percentage was 51.5% due to the thermal decomposition of the silk protein. Considerable attention was given to area II, which represents the structural transformation of the assembled SF fabric. The peak temperature increased from 314.9°C to 322.3°C. At area III, the small fragments decomposed into a gas, and the mass loss percentage was 29.3%.

### 3.5 Flushing stability of self-assembled (ALG/RSF)_n_ multilayers

Flushing provided better understanding of the stability of the fabrics in response to shear stress. The self-assembled (ALG/RSF)_n_ silicon wafers after 75% ethonal treatment were flushed in a laminar flow system. After 24 h, the thickness was measured using AFM, as shown in Fig 8. There was little change between the unflushed and flushed samples for the first 1.5, 3.5 and 5.5 bilayers. It should be noted that the gap between the unflushed and flushed samples widened at 7.5 and 9.5 bilayers. Nonetheless, there were no significant differences after the flushing, and the deposition remained stable in vitro. These results indicate that the self-assembled (ALG/RSF)_n_ SF fabrics would withstand blood flow in vivo. Flushing experiments with longer times will be performed in the future.

### 3.6 Bursting strength of the fabrics

**Fig 10:**
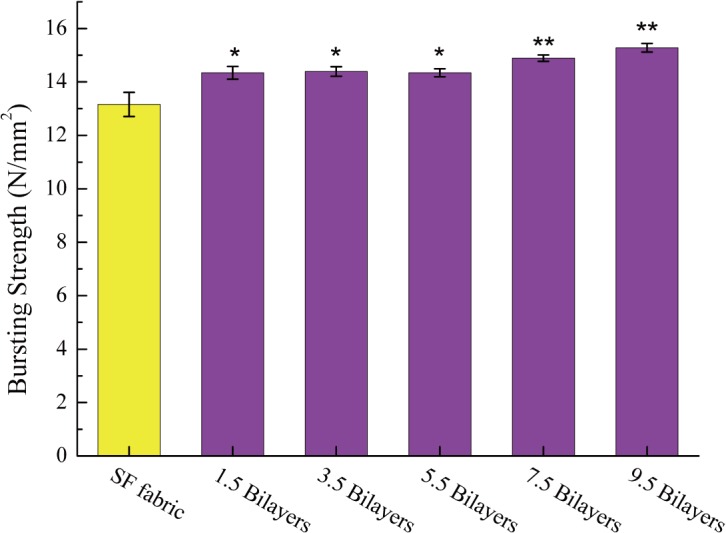
Bursting strength of SF fabric and self-assembled SF fabrics. (**) indicates significant differences of *p*<0.01 compared with SF fabric.

The bursting strength was measured to evaluate the mechanical properties of the SF fabric and self-assembled SF fabrics, and the results are shown in Fig 10. A difference was observed between the SF fabric and the self-assembled SF fabrics. The bursting strength of the SF fabric was 13.16±0.45 N/mm^2^. After the assembly processes in polyelectrolytes, the bursting strength increased markedly, and both strengths were greater than 14 N/mm^2^. The bursting strength of the (ALG/RSF)_9.5_ SF fabric was 15.28±0.16 N/mm^2^ and was 16.11% higher than that of the SF fabric.

On the one hand, an ALG/RSF deposition on the surface could bond with the warp and weft yarns, making the structure more tenacious. On the other hand, the crystallinity of the silk filament and assembled multilayers increased after treatment by 75% ethanol. Therefore, a mild modification of the environment will not influence the properties of the assembled SF fabrics. Such constructs were able to withstand more than 2500 mm Hg pressure (dog femoral and carotid arteries) [47].

### 3.7 Cell compatibility

**Fig 11:**
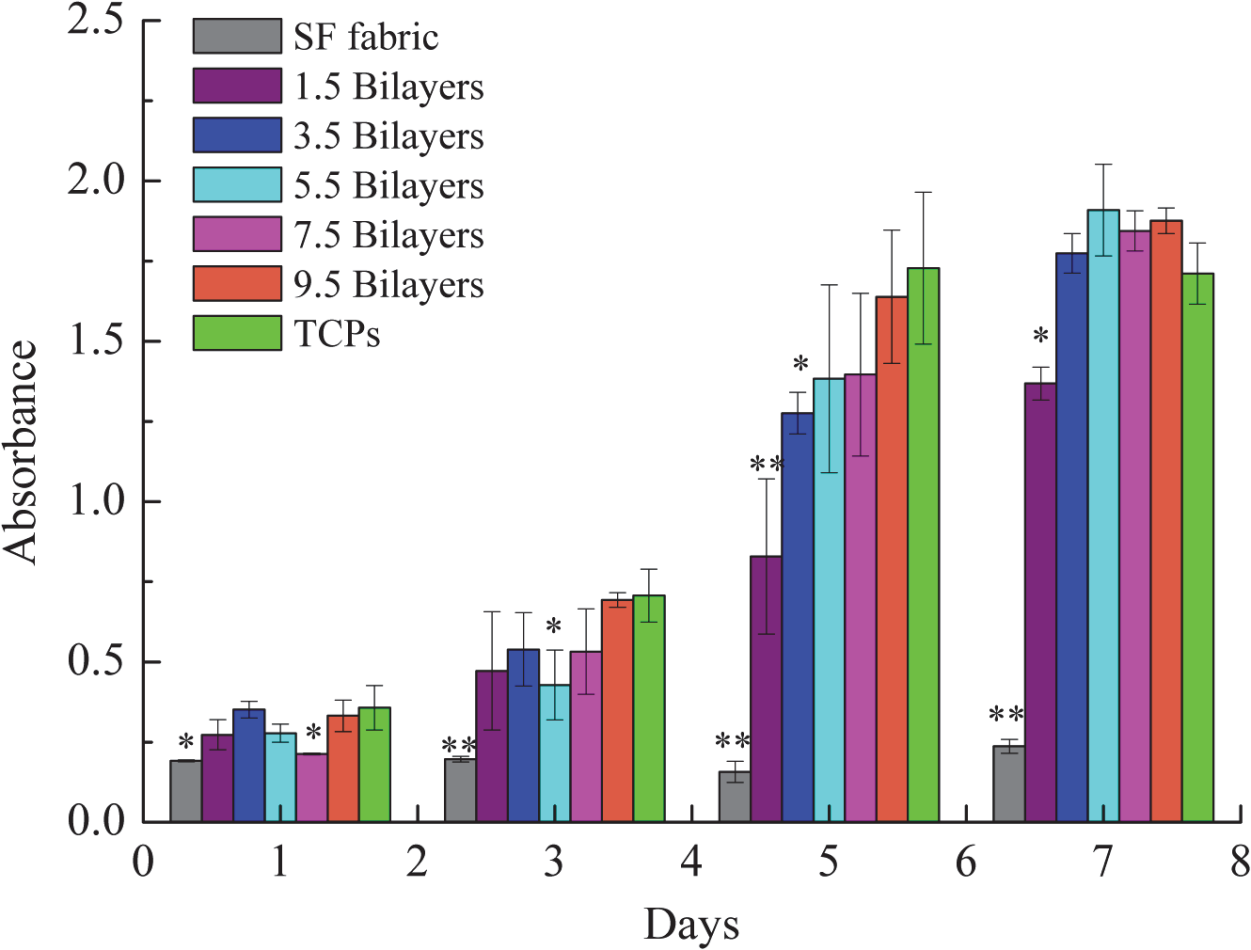
Proliferation of PIECs on SF fabric and (ALG/RSF)_n_ layer-by-layer self-assembled SF fabrics. Significant differences are marked by (*) for *p*<0.05 and (**) for *p*<0.01 compared with TCPs.

**Fig 12:**
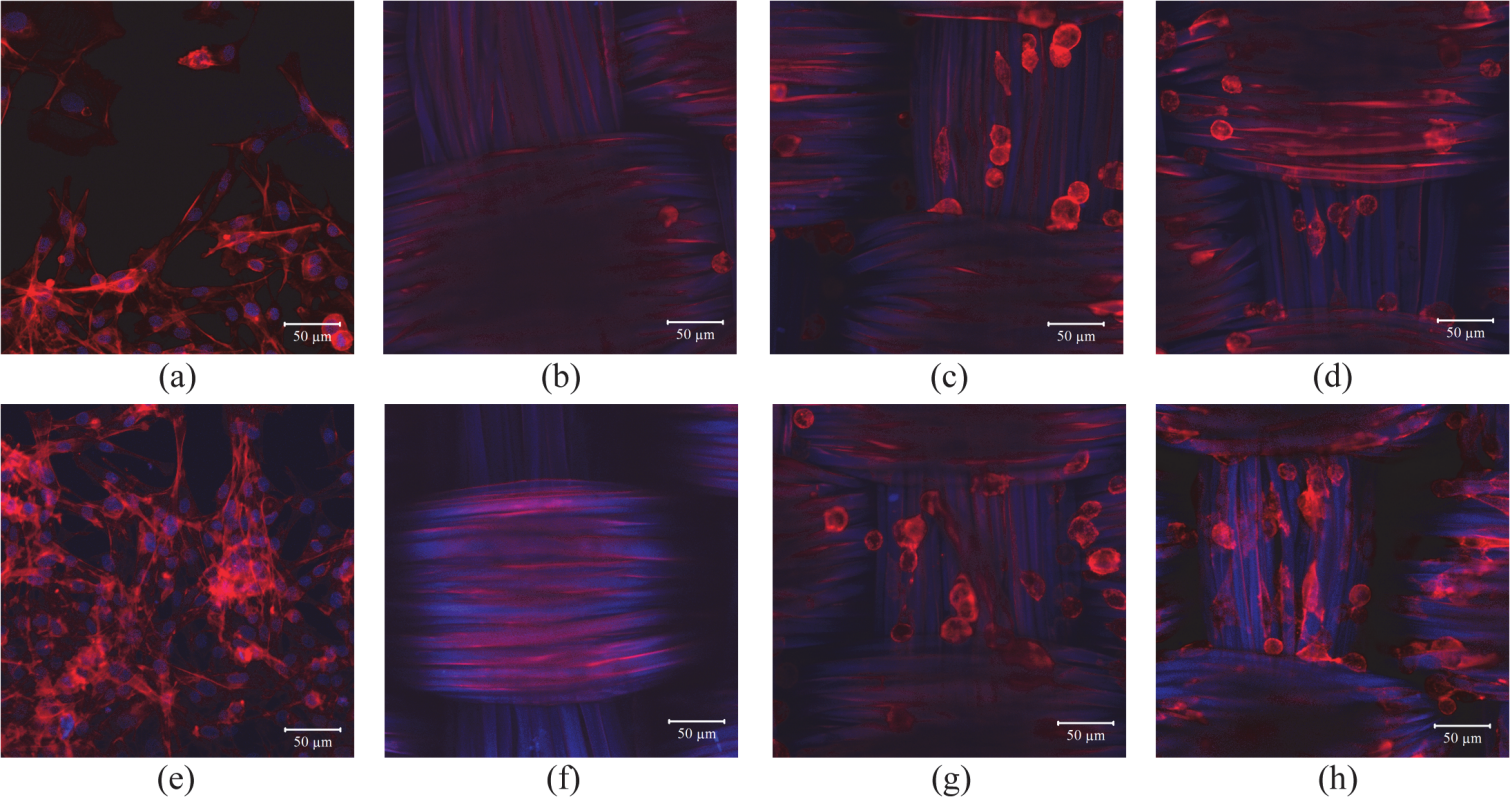
Confocal images of the PIECs on TCPs, SF fabrics and self-assembled (ALG/RSF)_n_ SF fabrics with the cell nuclei stained with DAPI (Blue) and F-actin stained with phalloidin-TRITC (Red). (a) TCPs cultured for 1 day, (b) SF fabric cultured for 1 day, (c) self-assembled (ALG/RSF)_**1.5**_ SF fabric cultured for 1 day, (d) self-assembled (ALG/RSF)_**9.5**_ SF fabric cultured for 1 day, (e) TCPs cultured for 3 days, (f) SF fabric cultured for 3 days, (g) self-assembled (ALG/RSF)_**1.5**_ SF fabric cultured for 3 days, and (h) self-assembled (ALG/RSF)_**9.5**_ SF fabric cultured for 3 days. The scale bar is 50 μm.

To assess the cell compatibility of the (ALG/RSF)_n_ layer-by-layer self-assembled SF fabrics, TCPs were selected as the positive control. A CCK-8 assay is shown in Fig 11 and reveals an increase in the PIECs on the self-assembled (ALG/RSF)_n_ SF fabrics. However, the SF fabric shows great toxicity and inhibits the cell proliferation. *S*. *C*. *Kundu* [48] reported that the silk fibroin coexist with sericin would trigger the inflammatory response. And the sericin cannot be removed totally. In contrast, the self-assembled (ALG/RSF)_1.5_ SF fabric shows mild toxicity. The surface was covered by depositions illustrated in Fig 2. After 7 days, the absorbance of the self-assembled (ALG/RSF)_n_ SF fabrics was higher than that of the TCPs. Compared with the TCPs, the (ALG/RSF)_n_ self-assembled SF fabrics had a 3D structure that could accommodate more cells, which can be confirmed in Fig 12.

The confocal images (Fig 12) of the fabrics indicated that the SF fabrics are not suitable for cell proliferation. There are barely any cells on the surface of the SF fabric (Fig 12(B) and 12(F)), demonstrating the poor biocompatibility of the SF fabric. After the (ALG/RSF)_n_ self-assembly, the cell compatibility obviously improved (Fig 12(C) and 12(D)). The cells proliferated as the culture time with the (ALG/RSF)_n_ self-assembled SF fabrics increased (Fig 12(G) and 12(H)). Moreover, the cells adhered to the surface of the fibres and spread along them.

It has been proved that surface roughness may affect cell attachment and proliferation[49]. *Lin*[50] has reported that 100 nm scale would increase cells adhesion and proliferation. Based on the AFM analysis in Fig 7, the distance between two adjacent peaks are calculated. It is 419 ± 145 nm for (ALG/RSF)_1.5_, 149 ± 38 nm for (ALG/RSF)_3.5_, 174 ± 75 nm for (ALG/RSF)_5.5_, 168 ± 55 nm for (ALG/RSF)_7.5_ and 102 ± 11 nm for (ALG/RSF)_9.5_. With the decreasing of the distance between two adjacent peaks, the cell proliferation improves obviously. It is 61.3%, 73.8%, 80.0%, 81.3% and 95.0% respectively compared with TCPs at fifth day in Fig 11.

### 3.8 Platelet adhesion on the SF fabric and self-assembled (ALG/RSF)_n_ SF fabrics

Circulating platelets in blood will adhere and form thrombi upon inflammation, thus damaging any implants [51, 52]. The dominant reaction during vascular graft implantation is platelet adhesion. Subsequently, endometrial hyperplasia can occur, leading to graft failure; therefore, platelet adhesion to vascular grafts must be monitored.

**Fig 13:**
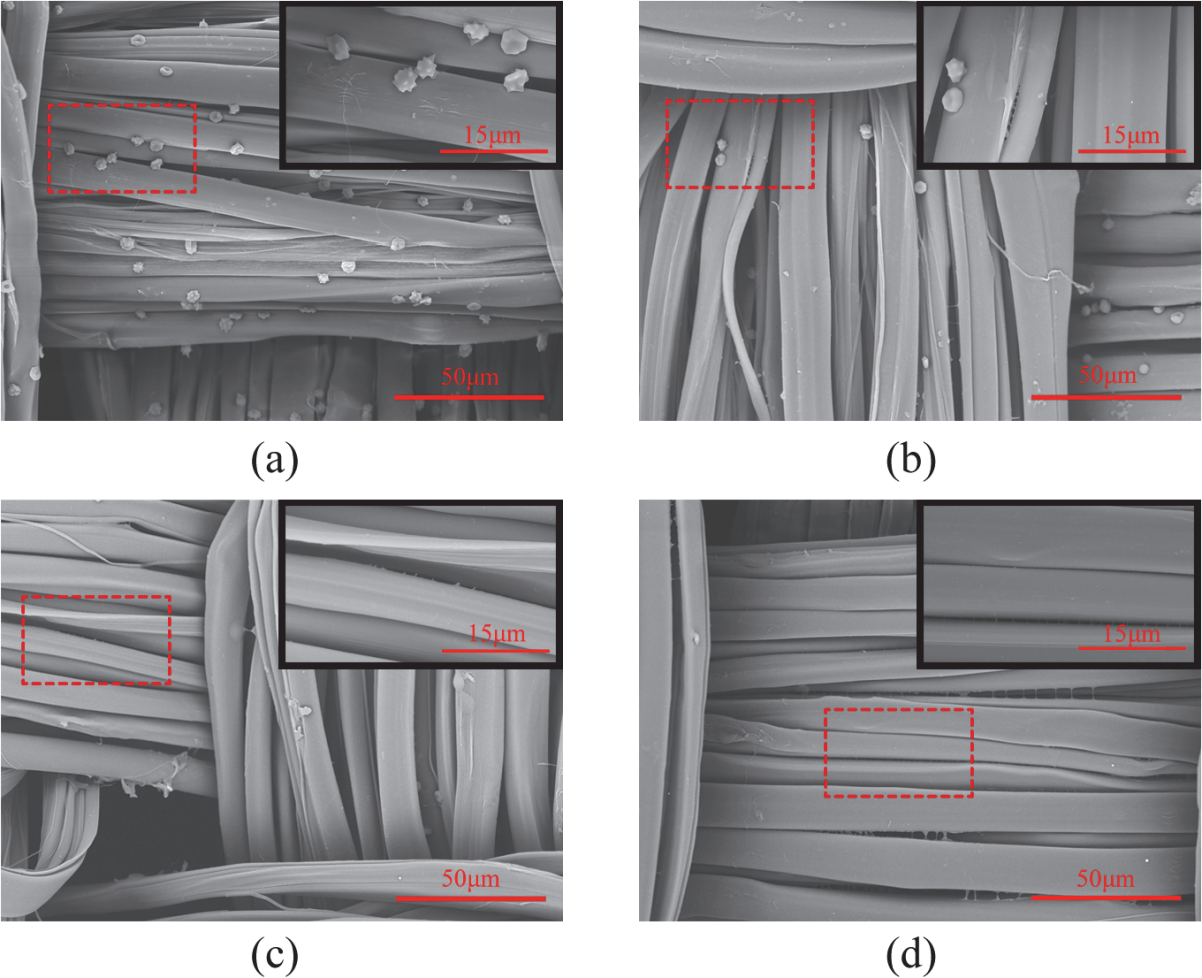
SEM photomicrographs of SF fabric and self-assembled (ALG/RSF)_n_ SF fabrics after exposure to a PRP/PPP mixture solution. (a) SF fabric, (b) self-assembled (ALG/RSF)_**1.5**_ SF fabric, (c) self-assembled (ALG/RSF)_**5.5**_ SF fabric, and (d) self-assembled (ALG/RSF)_**9.5**_ SF fabric.

Fig 13 shows the platelet activation and coagulation on SF fabrics and self-assembled (ALG/RSF)_n_ SF fabrics. The platelets coagulated in the surface of the SF fabric seriously. Moreover, pseudopods appeared after culture (Fig 13(A)). The assembly of 1.5 bilayers on the SF fabric markedly prevented the platelet adhesion (Fig 13(B)). There was little platelet adhesion and activation on the surface of the self-assembled (ALG/RSF)_1.5_ SF fabric. In addition, the self-assembled (ALG/RSF)_5.5_ and (ALG/SF)_9.5_ SF fabrics clearly showed almost no evidence of cellular adhesion (Fig 13(C) and 13(D)). The results of this limited in vitro experimental assay suggested that the self-assembled (ALG/RSF)_n_ multilayers of SF fabrics may be associated with a slower coagulation process. The repulsive force between platelets and a negatively charged surface is beneficial for preventing adhesion [53, 54].

### 3.9 Haemolysis assessment of fabrics

**Fig 14:**
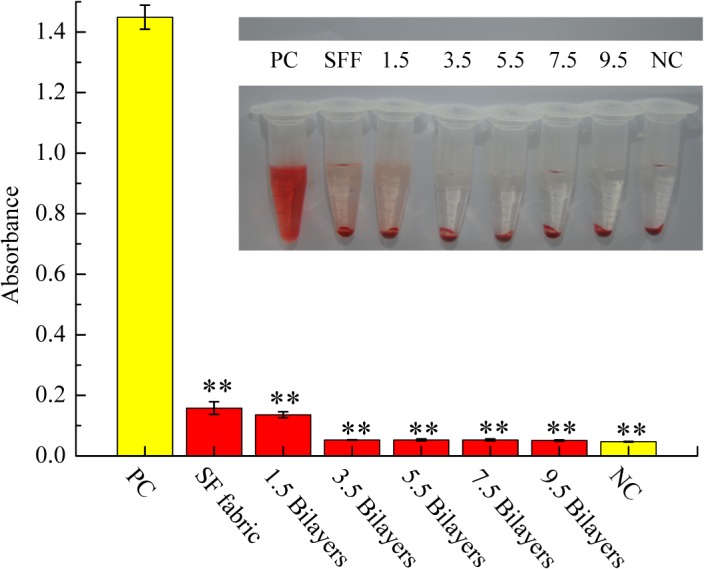
Mean haemolytic assay results. HRBCs exposed to the SF fabrics and self-assembled (ALG/RSF)_**n**_ SF fabrics (1.5, 3.5, 5.5, 7.5, and 9.5 bilayers) are compared with those exposed to PBS solution (negative control, NC) and water (positive control, PC). Mean data for each sample (n = 3). Significant differences are marked by (**) for *p*<0.01 compared with the positive control.

Haemolysis is a major problem associated with the destruction of red blood cells and the loss of cell function [40]. When red blood cells come into direct contact with foreign materials, such as vascular prostheses, prevention of haemolysis is important and necessary. The results of exposure of an HRBC suspension to the SF fabric and self-assembled (ALG/RSF)_n_ SF fabrics are shown in Fig 14. The positive control exhibited significant haemolytic activity, which was considered 100%; the negative control did not show any significant haemolytic activity, which was considered 0%. The results obtained with each of the fabrics are listed in Table 2. The SF fabric (HP% = 7.89±1.52%) and the 1.5 bilayers of self-assembled SF fabric (HP% = 6.35±0.69%) showed little haemolytic activity. As the number of bilayers increased, the haemolysis rate decreased obviously. There was no significant haemolytic activity, similarly to the negative control.

**Table 2:** Haemolysis percentage (HP) of SF fabric and (ALG/RSF)_n_ self-assembled SF fabrics.

Sample	SF fabric	(ALG/RSF)_1.5_	(ALG/RSF)_3.5_	(ALG/RSF)_5.5_	(ALG/RSF)_7.5_	(ALG/RSF)_9.5_
HP (%)	7.89±1.52	6.35±0.69	0.45±0.09	0.45±0.24	0.45±0.18	0.31±0.17

In the ASTM F756-00 standard practice, biomaterials are classified as non-haemolytic (0–2% haemolysis), slightly haemolytic (2–5% haemolysis), and haemolytic (>5% haemolysis). Our results showed that the SF fabric and the 1.5 bilayer self-assembled SF fabric were, according to this standard, classified as haemolytic, whereas the 3.5, 5.5, 7.5, and 9.5 bilayer self-assembled SF fabrics were non-haemolytic. The main reason for this difference was the residual sericin on the SF fabric. Natural silk fibres are spun together with a coating of sericin, which is known to damage red blood cells [55]. Therefore, natural SF has a tendency to be haemolytic when used as a blood-contacting material. Only the sericin that is located on the surface of an SF fabric can be completely removed by the degumming process; the sericin in the underlying zone and at the interlacing points of silk yarns cannot be thoroughly removed by degumming.

## Conclusion

In this study, alginate and silk fibroin were assembled on SF fabrics to create a biomaterial that could be used in a vascular graft. The outermost layer was negatively charged by SF deposition. The assembled SF fabrics were induced by 75% ethanol, which transformed the protein structure from random coils to β-sheets and made the modification of the SF fabric more stable. The thickness of the assembled multilayers increased exponentially. At the same time, the roughness of the surface improved as the number of assembled multilayers increased. The self-assembled SF fabrics were more stable under heating conditions. The (ALG/RSF)_n_ depositions combined together securely and were able to withstand flushing in a simulated in vitro environment. The bursting strengths of the assembled SF fabrics were higher than that of SF fabric and reached values greater than 14 N/mm^2^. The cytocompatibility significantly improved after the layer-by-layer self-assembly with ALG/RSF. An endothelial layer formed on the surface after 5 days of culture. A non-haemolytic classification (<2%) can be achieved by the self-assembly of more than 1.5 bilayers. The platelet adhesion results were better for the self-assembled (ALG/RSF)_5.5_ and (ALG/RSF)_9.5_ SF fabrics than for the SF fabric and the self-assembled (ALG/RSF)_1.5_ SF fabric. This research is the first to use both alginate and silk fibroin as polyelectrolytes in a layer-by-layer self-assembly procedure combined with 75% ethanol treatment. The experimental results indicate that the (ALG/RSF)_n_ layer-by-layer self-assembled SF fabric is an excellent biomaterial with improved properties compared with SF fabric. Future work will include subcutaneous implantation and animal experiments.
